# Pan-cancer deconvolution of tumour composition using DNA methylation

**DOI:** 10.1038/s41467-018-05570-1

**Published:** 2018-08-13

**Authors:** Ankur Chakravarthy, Andrew Furness, Kroopa Joshi, Ehsan Ghorani, Kirsty Ford, Matthew J. Ward, Emma V. King, Matt Lechner, Teresa Marafioti, Sergio A. Quezada, Gareth J. Thomas, Andrew Feber, Tim R. Fenton

**Affiliations:** 1 Department of Oncology, UCL Cancer Institute, University College London, London, WC1E 6BT UK; 2 Department of Haematology, UCL Cancer Institute, University College London, London, WC1E 6BT UK; 3 Cancer Sciences Unit, University of Southampton, Tremona Road, Southampton, SO16 6YD UK; 4 Department of Pathology, UCL Cancer Institute, University College London, London, WC1E 6BT UK; 5 Division of Surgery and Interventional Science, University College London, London, WC1E 6BT UK; 6 School of Biosciences, University of Kent, Canterbury, CT2 7NJ UK; 7 Present Address: Princess Margaret Cancer Centre, Toronto, ON M5G 2C4 Canada

## Abstract

The nature and extent of immune cell infiltration into solid tumours are key determinants of therapeutic response. Here, using a DNA methylation-based approach to tumour cell fraction deconvolution, we report the integrated analysis of tumour composition and genomics across a wide spectrum of solid cancers. Initially studying head and neck squamous cell carcinoma, we identify two distinct tumour subgroups: ‘immune hot’ and ‘immune cold’, which display differing prognosis, mutation burden, cytokine signalling, cytolytic activity and oncogenic driver events. We demonstrate the existence of such tumour subgroups pan-cancer, link clonal-neoantigen burden to cytotoxic T-lymphocyte infiltration, and show that transcriptional signatures of hot tumours are selectively engaged in immunotherapy responders. We also find that treatment-naive hot tumours are markedly enriched for known immune-resistance genomic alterations, potentially explaining the heterogeneity of immunotherapy response and prognosis seen within this group. Finally, we define a catalogue of mediators of active antitumour immunity, deriving candidate biomarkers and potential targets for precision immunotherapy.

## Abstract (web summary)

Determining the extent of immune cell infiltration into solid tumours is essential for adequate therapeutic response. Here the authors develop a DNA methylation-based approach for tumour cell fraction deconvolution and analyse tumour composition and genomics across a wide spectrum of solid cancers.

## Introduction

The tumour microenvironment plays key roles in shaping tumour evolution and in determining treatment responses; prominent intratumoural lymphocyte infiltration is a favourable prognostic marker in multiple tumour types, while a high stromal content of extracellular matrix-producing cancer-associated fibroblasts (CAF), is associated with poor outcomes^1^. The recent clinical success of immunotherapy in subpopulations of patients with previously intractable malignancies has also highlighted the importance of understanding the tumour microenvironment in order to identify those patients who will derive the most benefit from targeted therapies^1,2^. Although responses to immune checkpoint blockade (ICB), e.g. antibodies against PD-1 (programmed cell death protein 1), PD-L1 (programmed death-ligand 1) and CTLA-4 (cytotoxic T-lymphocyte-associated protein 4), are seen across many solid tumours, the proportion of patients that benefit varies widely by cancer type and we currently lack biomarkers with which to reliably predict immunotherapy response^3^. Emerging evidence from clinical trials indicates higher response rates in those cancer types that typically display greater lymphocyte infiltration (e.g. melanoma, lung cancer, head and neck cancer) and that the tumour neoantigen repertoire (a function of somatic mutation load) is a key determinant^4–7^. These observations point to a model in which, within any given cancer type, there are immune hot and immune cold tumours. Immune hot tumours display greater cytotoxic T-lymphocyte (CTL) infiltration, and reactivation of these tumour-resident CTLs by checkpoint inhibition can result in dramatic tumour regression. Conversely, immune cold tumours display minimal CTL infiltrates and typically fail to respond to checkpoint modulation. If one could accurately identify likely responders for patient stratification and devise strategies by which to convert cold tumours to hot tumours, these would be major steps forward in realising the full clinical potential of cancer immunotherapy.

Although flow cytometry of disaggregated tumour biopsies is commonly used for investigating cellular composition, this is often unfeasible for several reasons: difficulty in obtaining fresh tumour tissue; lack of defined markers for poorly characterised cell types (e.g. CAFs); and high cost of labour, reagents and equipment required for such analyses. Cellular disaggregation of collagen-rich tumours is also problematic, where cells are embedded in a dense extracellular matrix. To overcome these difficulties, multiple reference-free or reference-based methods have recently been developed to permit the in-silico deconvolution of complex cellular mixtures or to estimate tumour purity^8–15^. For example, accurate deconvolution of complex cellular mixtures, including tumours, has recently been achieved by the application of support vector regression modelling (CIBERSORT) to gene expression microarray data^14,16^. Notably, DNA methylation data are also suitable for deconvolution of tissue mixtures, although studies so far have focussed primarily on simple tissues such as blood, where cell type differences are a major confounder in Epigenome Wide Association Studies^10^.

Here, we present CIBERSORT-based deconvolution to genome-wide DNA methylation data from whole tumour tissue (hereafter referred to as ‘MethylCIBERSORT’). We provide accurate estimates of tumour purity and cellular composition, and identify immune hot and cold tumours across a broad spectrum of cancer types profiled by The Cancer Genome Atlas Project (TCGA). Using matched genomic and transcriptomic data, we identify multiple copy number alterations enriched in cold tumours, including deletions in *PTEN* and amplifications in *MYC* and *EGFR*. We show that responses to PD1-blockade are associated with a transcriptional signature for hot tumours post-treatment, while the cold signature, and specifically a gene expression module we previously linked to increased aerobic glycolysis downstream of EGFR in head and neck squamous cell carcinoma (HNSCC)^17^, is enriched in non-responders. Importantly however, defining whether a tumour is hot or cold is not sufficient to accurately predict response to ICB, and by interrogating matched genomic data, we show that treatment-naive hot tumours frequently display genomic alterations known to confer immunotherapy resistance.

## Results

### DNA methylation-based tumour deconvolution using CIBERSORT

**Fig. 1:**
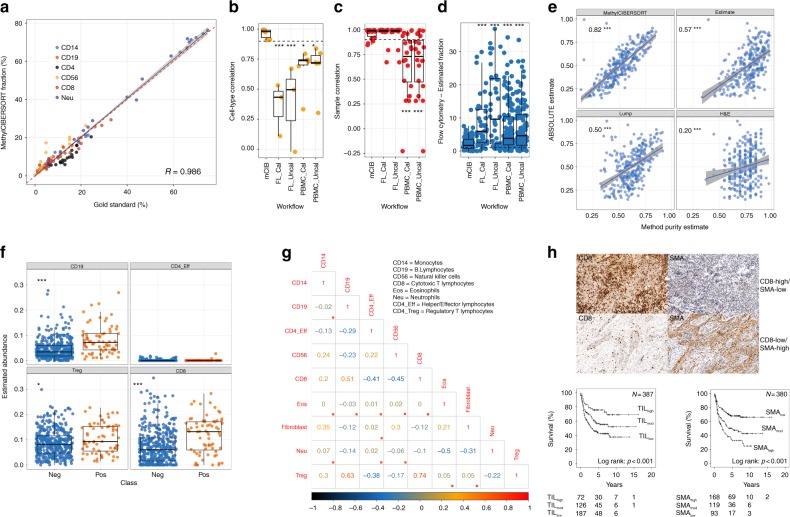
Validation of DNA methylation-based deconvolution for the analysis of tumour composition. **a** Correlation between MethylCIBERSORT fractions and flow cytometry for PBMC mixtures in independent data. **b**–**d** Boxplots showing comparisons between MethylCIBERSORT and flow cytometry versus Expression-CIBERSORT and flow cytometry in mixtures of similar complexity for correlations by cell type, correlations within samples, and finally absolute error. **e** Spearman’s correlations between ABSOLUTE and MethylCIBERSORT versus other previously published purity estimation methods. **f** Validation of previously reported associations between CD8 T-cells and B-cells and HPV status by HPV status. **g** Correlation plot showing Spearman’s Rho between cell-types in HPV− HNSCC, red boxes indicate nonsignificance at *q* < 0.1. **h** IHC showing a representative image of CD8 and SMA and Kaplan–Meier curves confirming the prognostic impact of TILs and fibroblasts in HPV-negative HNSCC. In boxplots, the ends of the boxes and the middle line represent the lower and upper quartiles, and medians, respectively. Whiskers represent 1.5 times the interquartile range (IQR)

To develop a DNA methylation-based deconvolution pipeline for application in tumours, we created a custom R interface to produce basis matrices for use with CIBERSORT and generated a reference using fibroblasts and seven different immune cell types (see Methods for details). We then evaluated the ability of our feature selection heuristic to accurately deconvolute mixtures of leukocytes using publicly available methylation data from mixtures of peripheral blood mononuclear cells (PBMCs) with composition verified by flow-cytometry (gold standard). This showed an extremely high correlation between the estimated and gold-standard fractions (Pearson’s *R* = 0.986, *p* < 2.2e−16, Fig. 1a). We also carried out benchmarking against the performance of RNA-based CIBERSORT using the LM22 basis matrix against leukocyte mixtures of similar resolution originally profiled in Newman et al.^14^. This revealed that MethylCIBERSORT estimates demonstrate higher correlations, both at the cell-type and the sample level (Fig. 1b, c) and significantly lower absolute error (Fig. 1d). Thus, methylation data coupled to CIBERSORT is highly accurate and may offer distinct advantages relative to expression-based CIBERSORT.

To validate the method on real tumour samples, especially the tumour content in order to permit absolute quantification of tumour composition, we focused initially on HNSCC, a tumour type in which we have previously demonstrated the prognostic significance of tumour-infiltrating lymphocytes (TILs), particularly in those cancers driven by human papillomavirus (HPV)^18,19^. We applied our pipeline to generate an HNSCC-specific basis matrix and applied it to the set of 464 HNSCCs that have both RNA-sequencing and DNA methylation profiles available from TCGA^20^. Upon comparing cancer cell proportion (purity) estimates derived using MethylCIBERSORT with estimates derived from ABSOLUTE^21^ (which jointly estimates purity and ploidy using mutation and copy number data) relative to other previously published methods of estimating purity (LUMP^22^ and ESTIMATE^23^) with data aggregated in ref. ^22^, MethylCIBERSORT displayed the highest correlation (*R* = 0.82) and better concordance with ABSOLUTE than other methods (Fig. 1e). Analysis of residuals (method estimate—ABSOLUTE estimate) suggested close concordance with ABSOLUTE estimates for MethylCIBERSORT, with larger deviations only seen when samples were of very high purity (>80%), while other methods tended to overestimate tumour cell content in samples of low purity (Supplementary Figure 1a), resulting in statistically significant differences in distributions (FDR < 2.2e−16).

We also compared the mRNA expression of a panel of cellular lineage markers with MethylCIBERSORT estimates and found significant associations for multiple cell types (Supplementary Figure 1b) even though they are derived from different samplings of the same tumour. Many of these marker genes demonstrated more variable expression in tumours with lower estimates of infiltrating cell fraction, suggesting that low coverage on either or both platforms (RNA-seq and methylation array) at the lower end of cellular abundance may result in poorer concordance. Taken together, these observations confirm that MethylCIBERSORT can accurately deconvolute the mixed cell populations in tumour samples using DNA methylation data.

Having established the potential of MethylCIBERSORT to identify patterns of cellular infiltration in solid tumours, we tested its ability to detect the elevated TIL levels previously documented in HPV-driven (HPV+) HNSCC^19^. MethylCIBERSORT detected not only the increased TIL levels in HPV+ HNSCC compared with HPV− HNSCC (*p* = 2.167e−05, Wilcoxon’s Rank Sum Test) but more specifically attributed this to increased numbers of B (CD19+) and cytotoxic T (CD8+) lymphocytes (CTLs, Fig. 1f), in agreement with observations made using other methods, including immunohistochemistry and gene expression analysis^24^, potentially also helping to explain favourable prognosis displayed by this subgroup, independent of treatment modality^25–27^.

### Cellular infiltration patterns in HPV-negative HNSCC

Next, we extended our analysis to HPV-negative (HPV−) HNSCC, a heterogeneous, anatomically-diverse group of tumours in which prognosis is typically much poorer than in HPV+ disease. Again, using TCGA data (available for 398 HPV− HNSCCs) we observed interesting relationships between multiple cell types, with 24/36 pairs of cell types showing significant correlations (Spearman’s rank correlations, FDR < 0.1; Fig. 1g). CTLs are associated with both CD14+ (monocytes/macrophages/MDSCs) and B-lymphocytes (Rho = 0.2 and 0.51). CD4+/FoxP3− T-lymphocytes (CD4+ effector T-lymphocytes), meanwhile display inverse correlations with CTLs (*R* = −0.41) and Tregs (*R* = −0.38). CD56+ natural killer (NK) cell abundance is also inversely correlated with CTLs (*R* = −0.45). Of note, CTLs are inversely correlated with fibroblast abundance (*R* = −0.12) and to validate this latter finding, we analysed data from two large studies in which these parameters had been quantified in HNSCC^19,28^. In a pooled analysis of these data, TIL content and SMA expression (a CAF marker) are inversely correlated (*r* = −0.322 and −0.344 for CD8 and CD3 IHC in the Ward (oropharyngeal SCC) cohort (Fig. 1h); −0.4 and −0.424 for TIL scoring of H&E sections in the Ward (oropharyngeal SCC) and Marsh (oral SCC) cohorts, respectively). They are also strongly prognostic (Fig. 1h; *p* < 0.001, Log Rank Test).

**Fig. 2:**
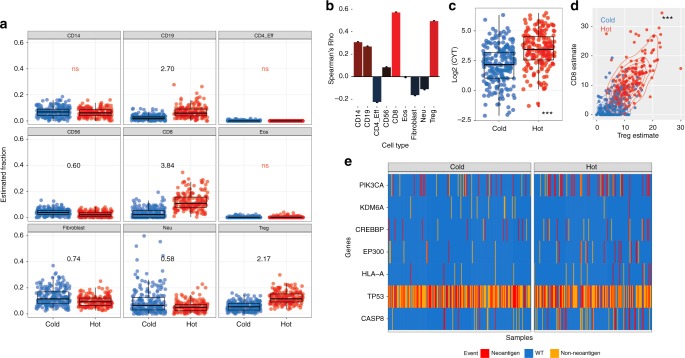
Classification of HNSCC into hot and cold subgroups on the basis of immune cell infiltration patterns. **a** Boxplot of cell-types based on clustering HNSCC. Relative abundance of each cell type in hot versus cold tumours is indicated as a fold-change on each plot for all significant differences (*q* > 0.05, Wilcoxon’s Rank Sum Test with BH correction for multiple testing) except where indicated (ns). **b** Bar-graph showing associations between cytolytic activity and cell types. **c** Cytolytic activity is elevated in immune-hot HNSCC. **d** CD8/Treg ratios by HNSCC immune cluster. **e** Mutations significantly associated with HNSCC immune cluster. In boxplots, the ends of the boxes and the middle line represent the lower and upper quartiles, and medians, respectively. Whiskers represent 1.5 times the interquartile range (IQR)

Given the complex nature of associations between different cell types, we performed consensus PAM clustering on the estimated cellular fractions to define subgroups by infiltration patterns. We derived two clusters (immune cold and immune hot, hereafter referred to as cold and hot, respectively) that show markedly different distributions of multiple cell types, most notably CTLs, Tregs, CD4+ effector T-lymphocytes, CD19+ B-lymphocytes and NK cells, all of which are implicated in antitumour immunity (Fig. 2a). Consistent with our previous observations, estimates of fibroblast content are higher in the cold group (mean fold change 1.35, FDR < 5e−7, Wilcoxon’s Rank Sum Test). To explore the functional significance of our observations, we tested for associations between individual cellular fractions or immune cluster and a recently defined measure of local cytolytic activity based on the expression of Granzyme A and Perforin 1 (*GZMA* and *PRF1*; markers of activated T-cells)^29^. Most infiltrating cell fractions display significant correlations with cytolytic activity, with CD8+ cells showing the maximum positive correlation (Fig. 2b, FDR < 0.05, Spearman’s Rank Correlation). Accordingly, the hot cluster displays significantly higher cytolytic activity (Fig. 2c, *p* = 2e−16, Wilcoxon’s Rank Sum Test), and increased ratios of CTLs to Tregs (Fig. 2d, *p* < 2e−16, Wilcoxon’s Rank Sum Test); a metric that is prognostic in multiple settings^30–32^.

Together, our analyses suggest a tilting of the balance towards CTL activity in the microenvironment of tumours from the hot cluster. Integrated analysis of the impact of different cell populations on cytolytic activity using linear modelling identified CD8+ (coef = 0.13, *p* < 2e−16), CD14+ (coef = 0.10, *p* < 7e−7), and CD56+ (coef = 0.26, *p* < 5e−15) cell abundance as positive predictors and fibroblast abundance as a negative predictor (coef = −0.08, *p* < 1e−10).

### Transcriptional and proteomic differences between hot and cold HNSCCs

Having observed differential abundance of various leukocytes in hot versus cold HNSCC using MethylCIBERSORT, we used gene expression data to further validate these estimates. Using limma-trend analysis, we identified 458 differentially expressed genes (DEGs) between the hot and cold clusters at a fold-change of greater than 2 (FDR < 0.01, Supplementary Data 1, genes highlighted in bold). Multiple DEGs are consistent with the MethylCIBERSORT-derived estimates of lymphocyte infiltration; *CD8A*, *ZAP70* and *CD3D* (CD8 lymphocyte markers), *CD79A* and *CD19* (B-lymphocyte markers), are all upregulated in the hot tumours, as are multiple chemokines and their receptors (*CCL5*, *CCR5*, *CXCR5*, *CXCR6*, *CCL19*, *CXCL11*), immune checkpoint gene transcripts (*LAG3*, *PD1*, *IDO1*), and as expected, the cytolytic markers *PRF1* and *GZMA*. In extended analyses of all genes at FDR < 0.01 (Supplementary Data 1), multiple other genes, including the Class 1 MHC gene *B2M* (FC = 1.39), PD-1 ligand *CD274* (FC = 1.62) and *ACTA2*, which encodes SMA (FC = 0.68), are also differentially expressed between the two clusters, the latter validating fibroblast estimates from MethylCIBERSORT (Fig. 2a).

Ingenuity Pathway Analysis further confirmed observations made using MethylCIBERSORT estimates, identifying differential regulation of multiple canonical pathways associated with immune function and inflammatory conditions (Supplementary Data 2), consistent with differential lymphocyte infiltration and activity. Diseases and functions ontology (Supplementary Data 3) indicated that the top few pathways activated in hot tumours were associated with leukocyte and lymphocyte migration. Upstream regulatory analysis implicated increased activation of STAT1 and IRF1, and inhibition of Interferon-stimulated transcription mediated by IRF4, in hot tumours (Supplementary Data 4). Finally, analysis of RPPA data identified 10 differentially abundant (FDR < 0.1) proteins or phospho-proteins (Supplementary Data 5). Higher levels of cleaved Caspase 7 (FC = 1.52) in the hot subgroup indicates increased apoptosis, whereas Fibronectin and PAI1 upregulation in immune cold tumours suggest a distinct pattern of TGFβ-driven extracellular matrix remodelling in what may be a CAF-linked phenomenon.

### Distinct mutations are associated with HNSCC immune cluster

Having established that the two immune clusters display distinct transcriptional patterns, we then sought to identify individual mutations in driver genes (MutSigCV^33^ *q* value < 0.01) associated with immune cluster using negative binomial regression. This identified enrichment of *CASP8*, *PIK3CA*, *CREBBP*, *EP300* and *HLA-A* mutations in hot HNSCCs, while *TP53* and *KDM6A* mutations are enriched in cold HNSCCs (Fig. 2e). *CASP8* mutations are implicated in subverting apoptosis induced by lymphocytes; they are enriched in tumours with high immune cytolytic activity and likely reflect an increased selective pressure exerted by the presence of adaptive immune cells^29,34^. Fas-ligand (*FASLG*), an upstream activator of pro-apoptotic signalling through Caspase 8 is also upregulated in the immune hot tumours, further highlighting the importance of this pathway (Supplementary Data 1). Identification of this lymphocyte-rich group displaying *CASP8* mutations and a relative lack of *TP53* mutations is striking, since TCGA previously identified a subset of good-prognosis oral cavity tumours bearing the same genomic hallmarks, which were reported to co-occur with *HRAS* mutations^20^.

Neoantigen burden has previously been identified as a predictor of anti-tumour immune responses^29,35,36^ and consistent with this, we identified significantly higher predicted MHC Class I neoantigen burdens in the hot tumours (OR = 1.56, *p* < 5.5e−8, negative binomial GLM) and a smaller increase in overall mutational burden (OR = 1.46, *p* = 6e−7). Moreover, in 15 tumours from the hot cluster versus five in the cold cluster, *CASP8* mutations themselves encoded at least one neoantigenic peptide (Fig. 2e), demonstrating the existence of mutations that could both contribute to the development of a potential selective constraint, and serve as an adaptive mechanism to evade it.

### Deconvolution and immune clustering across tumour types

To examine whether the relationships between tumour composition and genomic alterations we observed in HNSCC are generally applicable, we derived cancer-type specific basis matrices and conducted deconvolution on 18 further tumour types for which cancer cell line methylation data have recently been published^37^. For nine of these we were able to compare our predictions of tumour purity with ABSOLUTE estimates and observed strong correlations and significantly lower error margins compared to LUMP and ESTIMATE (Supplementary Figure 2a, b). Further, we observed a robust preservation of positive correlations between MethylCIBERSORT and marker expression pan-cancer (Supplementary Figure 2c), again with the caveat that the samples were taken from different aliquots of the tumour. Taken together, these findings attest to the general pan-cancer applicability of MethylCIBERSORT. An important potential advantage of DNA-methylation over gene expression-based deconvolution methods is the ease with which accurate DNA methylation profiles can be obtained from formalin-fixed, paraffin-embedded (FFPE) samples^38^. We therefore compared estimates pertaining to fresh frozen and matched FFPE samples (*n* = 21 from three tumours)^39^ and recorded very high correlations, indicating our method is applicable also to the archival material (Supplementary Figure 2d).

**Fig. 3:**
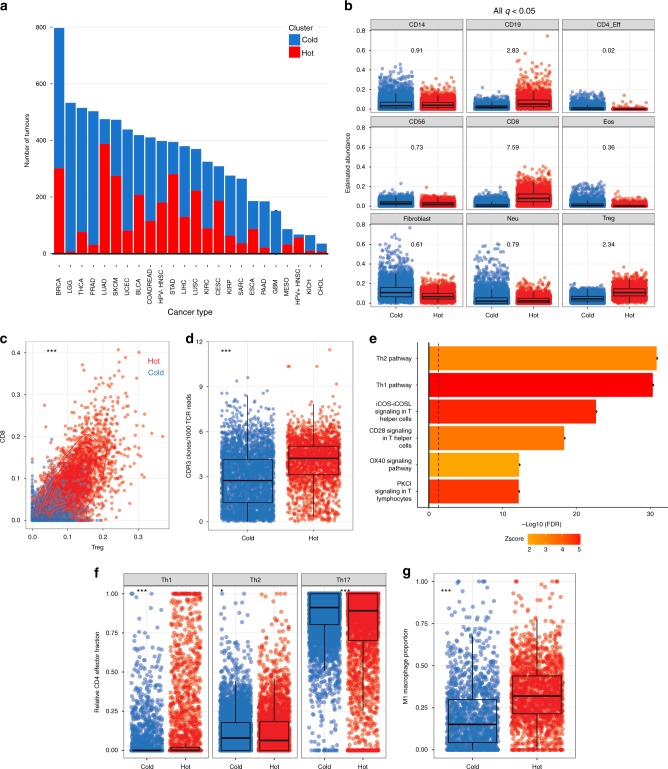
Identification and characterisation of hot and cold tumours pan-cancer. **a** Barplot of distribution of immune-hot and cold tumours across TCGA. Cancers known to respond favourably to checkpoint blockade, such as lung cancer and melanomas, show high fractions of hot tumours. **b** Boxplot of cell-type estimates by immune cluster. All at *q* < 0.05. Numbers represent mean fold changes. **c** CD8:Treg ratio is elevated in hot tumours pan-cancer. **d** Increased breadth of TCR sequences in immune hot tumours (Wilcoxon’s Rank Sum Tests). **e** Results of IPA canonical pathway analysis comparing hot and cold tumours pan-cancer after adjusting for tumour type. **f** Transcriptional deconvolution by expression-based CIBERSORT shows immune cluster (*x*-axis) is associated with distinct CD4 polarisation and **g** macrophage polarisation (CIBERSORT fractions on *y*-axis). *p* Values are from Wilcoxon’s Rank Sum Tests. In boxplots, the ends of the boxes and the middle line represent the lower and upper quartiles, and medians, respectively. Whiskers represent 1.5 times the interquartile range (IQR)

We then trained an elastic-net classifier using 5-fold cross-validation for tuning on the HNSCC cellular abundance data, returning highly accurate recapitulation of clustering (Kappa = 0.9), and predicted immune cluster membership for the validation set of 7596 samples representing 21 further tumour types from TCGA (Fig. 3a). As expected, we observed strong enrichment for CTLs, Tregs and B-lymphocytes in hot tumours pan-cancer, while CD4-effectors, NK cells, eosinophils and CAFs were enriched in immune cold tumours (Fig. 3b). Different tumour types also display markedly varying degrees of lymphocyte infiltration, with the majority of pancreatic ductal adenocarcinomas, colorectal, thyroid, uterine corpus endometrial, kidney, prostate, hepatocellular cancers and sarcomas belonging to the cold cluster (Fig. 3a). We again observed increased CTL:Treg ratios in hot tumours (Fig. 3c) and similar relationships between tumour composition and CYT to those seen in HNSCC.

### Increased immunoediting and Th1/M1 responses in hot tumours

To further determine if the immune infiltrate was active in these tumours, we assayed immunoediting by testing for reductions from the expected ratio (as previously defined by Rooney et al.^29^) of observed neoantigens to total nonsilent mutations per tumour and adapted this approach to derive the estimated number of neoepitopes lost through immune editing while controlling for tumour type. Accordingly, we found significant enrichment for editing in hot tumours compared to cold tumours (OR = 1.28, *p* = 0.001, negative binomial GLM). Additionally, upon integration with T-cell receptor (TCR) repertoire data from Li et al.^40^, we found more diversity (Number of TCR clones/Total number of TCR reads) in the immune hot tumours (Fig. 3d, *p* < 2.2e−16, Wilcoxon’s Rank Sum Test), suggesting that broader immune responses may underlie the greater depletion of neoantigens in this group.

Given the evidence for divergent infiltration patterns and activity between the immune clusters across cancer types, we then investigated the determinants of this response by identifying DEGs after adjusting for tumour type. We identified 365 genes at FDR < 0.01, FC > 2 and in pathway analysis, the top pathways were significantly associated with T-helper 1 (Th1) versus T-helper 2 (Th2) lymphocyte responses (Fig. 3e, Supplementary Data 6). Multiple Th1 cytokines and downstream targets were overexpressed in hot tumours (*IFNG*, *CCL4*, *CCL5*, *CXCL9*, *CXCL10*), along with costimulatory and coinhibitory receptors, suggesting these tumours were marked by a state of lymphocyte activation and counter-responses thereto. We next scored proinflammatory (Th1, Th17) and suppressive (Th2) CD4+ cell populations using RNA-seq reference profiles from purified cells to derive relative estimates using CIBERSORT^14^. Consistent with our inferences from pathway analysis, we found enrichment for Th1 cells in hot, and Th2 and Th17 cells in cold tumours (Fig. 3f). Th2 cells have been linked to poor prognosis in multiple studies, while Th1 cells are associated with good prognosis and aiding CTL responses^1^.

We also used expression-based CIBERSORT to derive estimates for different myeloid cell populations (*n* = 2346 tumours at deconvolution *p* < 0.05, Permutation Test, 1000 replicates), and identified substantially higher fractions of M1 relative to M2 macrophages in hot tumours (*p* = 2.2e−16, Wilcoxon’s Rank Sum Test, Fig. 3g). Notably, M2-like polarisation is associated not only with Th2 immune responses but also with immune-suppressive myeloid-derived suppressor cells (MDSCs)^41^. Taken together, our analyses implicate Th1 cytokine signalling programmes as responsible for establishing an immune-hot state and suggest MDSC and Th2/Th17 programmes as targets for efforts to switch cold tumours to an immune hot state.

### Relation of immune cluster to ICB response

**Fig. 4:**
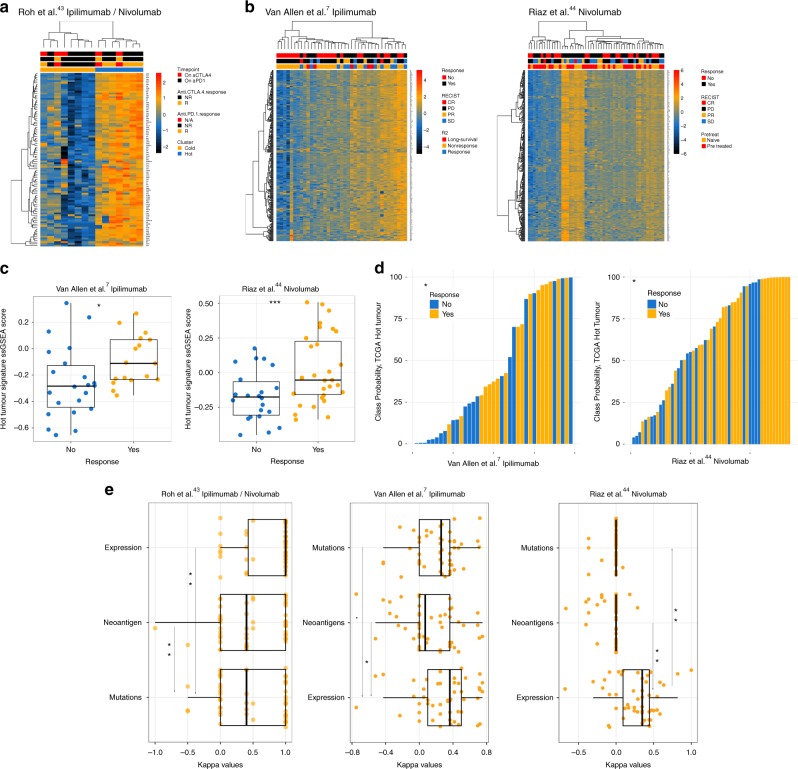
The immune-hot signature is associated with but not predictive of response to ICB in melanoma. **a** Heatmap showing expression of the hot-tumour transcriptional signature in Nanostring data from posttreatment biopsies of immunotherapy patients. **b** Heatmaps showing the same signature in RNAseq data of aCTLA4 (pre-treatment) and aPD1 (post-treatment), respectively. **c** Boxplots highlighting significant differences in ssGSEA scores for the hot-tumour transcriptional signature in the datasets featured in **b** (*p* values from logistic regression). **d** Barplots display similarity to TCGA hot and cold tumours based on logistic regression class probabilities from a model fit to TCGA data, which are associated with response. **e** Boxplots showing Kappa values from cross-validation for models examining the performance of the Immune-hot signature, Class I neoepitope burden, and finally mutational load on immunotherapy response classification (*p* values from Wilcoxon’s Rank Sum Test). In boxplots, the ends of the boxes and the middle line represent the lower and upper quartiles, and medians, respectively. Whiskers represent 1.5 times the interquartile range (IQR)

We reasoned that if the signature for immune hot tumours represented active immunity, it could be applicable to the prediction of immunotherapy responses and evaluated this hypothesis using tumour gene expression data from three melanoma cohorts: post-sequential aCTLA4 and aPD1 treatment^42,43^; pre-aCTLA4 treatment^7^ and post-aPD1 (Nivolumab) treatment^44^. Analysis of these transcriptional patterns indicated differential expression between responders and non-responders (Fig. 4a), and accordingly, ssGSEA scores for the hot transcriptional signature showed significant enrichment in responders for the latter two datasets (Fig. 4b, c). Moreover, a similar association emerged from comparing the probability of response to hot/cold class prediction, inferred using a logistic regression fit on TCGA hot/cold transcriptional signature ssGSEA scores (Fig. 4d). Finally, we evaluated the ability of the hot-signature to stratify patients by response relative to mutational load and Class I neoepitope burden using elastic nets coupled to cross-validation for each dataset (Fig. 4e). While larger patient cohorts will be required to search for and validate predictive ICB biomarkers, from this limited analysis it is clear that neither cellular composition as described by hot/cold classification, nor total mutation or predicted Class 1 neoantigen loads (which are also associated with response and have been proposed as ICB response biomarkers) are reliable predictors.

Our analysis clearly indicates there is a heterogeneity of ICB response within both hot and cold tumours similar to that described for tumours with high or low mutational loads, underlining the importance of additional factors not captured by these metrics, such as immune cell phenotype and the spatial distribution of immune cells within the tumours^1^. We reasoned that in addition to these factors, the heterogeneity in responses to ICB among hot tumours might be driven by intrinsic resistance to T-cell-mediated destruction due to pre-existing genomic alterations within the tumour cells. We set out to test this hypothesis by constructing a pan-cancer catalogue of genomic alterations enriched in hot tumours, with the additional aim of finding those alterations enriched in cold tumours which might drive lymphocyte exclusion or reduce tumour immunogenicity.

### Genomic features of hot and cold tumours

**Fig. 5:**
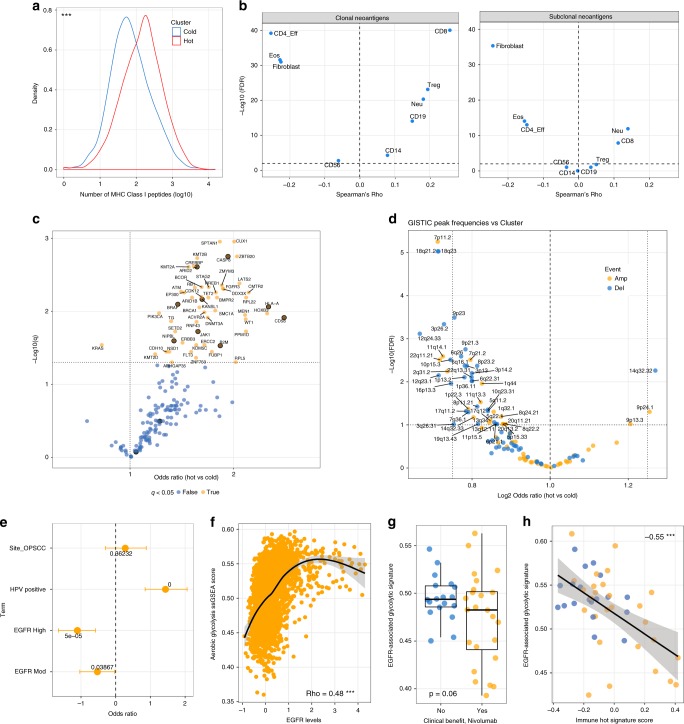
Genomic features of hot and cold tumours. **a** Density plots showing differences in neoantigen burden by immune cluster pan-cancer. *p* Value from negative binomial regression that accounts for tumour type. **b** Clonal neoantigens and subclonal neoantigens are correlated with different infiltration profiles. Volcanoplot shows Spearman’s Rho on the *x*-axis and −log10(FDR) on the *y*-axis. **c** Volcanoplot showing results of binomial regression testing for associations between immune-hot cancers and mutation frequencies in candidate cancer driver genes. Those genes implicated in resistance to T-cell-mediated killing (OR = 11.75, *p* < 0.0004, Fisher’s Exact Test, see Results text for details) are highlighted in orange. **d** Volcanoplot showing associations between GISTIC candidate driver copy number peaks and immune cluster. **e** Plot showing results of logistic regression in a cohort of HNSCCs where the probability of being classified TIL-high was regressed against anatomic subsite, EGFR IHC (low/moderate/high) and HPV status. **f** Correlation between glycolytic coexpression signature ssGSEA scores and EGFR levels by RPPA. **g** Association of glycolytic signature post-Nivolumab with response and **h** inverse correlation between the glycolytic signature and the immune-hot expression signature, Spearman’s correlation has been plotted. In boxplots, the ends of the boxes and the middle line represent the lower and upper quartiles, and medians, respectively. Whiskers represent 1.5 times the interquartile range (IQR)

Consistent with our observations in HNSCC, and with estimates from gene expression-based deconvolution^13^, immune hot tumours harboured higher overall mutation loads (OR = 1.33, *p* = 3e−25, negative binomial GLM controlling for cancer type) and more predicted neoantigens than immune cold tumours (Fig. 5a). Given the recent finding that in addition to the presence of neoantigens, their clonality (i.e. presence in all tumour cells as opposed to minor subclones) is associated with prognosis and response to Pembrolizumab in lung adenocarcinoma^5^, we analysed immune microenvironment composition as a function of neoantigen clonality (as denoted by The Cancer Immunome Atlas^45^). We found that the abundance of both CTLs and Tregs is correlated with clonal neoantigen load pan-cancer, while the relationship is much weaker when subclonal neoantigens are considered. CD4+/FOXP3− effector lymphocytes display a striking inverse correlation with clonal neoantigens (Fig. 5b). Consistent with our earlier observation that they are enriched in CTL/Treg low cold tumours, CAFs are inversely correlated with both clonal and subclonal neoantigen loads. Hot tumours display a significantly higher clonal neoantigen burden (OR = 1.28, *p* < 4e−7, negative binomial GLM) as well as a skew in the neoantigen burden towards clonal neoantigens after adjusting for tumour type (OR = 1.01, *p* = 0.006 negative binomial GLM). These findings provide evidence for a direct link between Class I MHC clonal neoantigen burden and patterns of TIL abundance and may help to explain the observations of McGranahan and colleagues, that high clonal neoantigen burden predicts favourable response to immune checkpoint modulation using Pembrolizumab^5^.

Next, we examined if the genomic features associated with immune cluster were also reproducible across cancer types, performing adjusted binomial regressions to estimate cluster association after controlling for tumour type for genes previously implicated as pan-cancer drivers based on signatures of positive selection^46^ and recorded 56 hits at FDR < 0.05 (Supplementary Data 7 and Fig. 5c). Interestingly, these putative drivers of hot tumours were significantly enriched (OR = 11.75, *p* < 0.0004, Fisher’s Exact Test) in a list of genes demonstrated to confer resistance to CD8 T-cell-mediated killing in a recent CRISPR-Cas9 screen^47^. The functionally-verified immune-resistance genes that were disproportionally mutated in hot tumours included those involved in antigen presentation (*B2M* and *HLA-A*), apoptosis (*CASP8*) and interferon signalling (*JAK1*). *ARID2*, which encodes a component of the SWI/SNF chromatin remodelling complex also implicated in modulating sensitivity to T-cell-mediated killing downstream of JAK-STAT signalling, was also disproportionately mutated in hot tumours^48,49^. Mutant *KRAS*, recently implicated in generating a fibrotic tumour microenvironment by activating paracrine signalling with CAFs in pancreatic cancer^50^, is enriched in cold tumours. Taken together, these findings support a model wherein mutations in certain genes render tumours hot as a consequence (and therefore susceptible to checkpoint blockade), or may enable tumours to survive in a hot tumour microenvironment, potentially also bestowing resistance to checkpoint blockade. We sought to test this model by linking our candidate immune-resistance mutations to lack of ICB response in pre-treatment hot tumours and although we observed a trend, (OR = 0.13, logistic regression, *p* = 0.09), the number of treated tumours with sequence data available is currently too low (54 hot tumours across four studies) to gain a definitive answer.

To complement these analyses, we called copy number alterations across 11,000 tumours and tested for differential association of peaks with immune cluster after adjusting for tumour type for the subset with immune cluster assignment available. This led to the identification of multiple events that occurred at different frequencies between cold and hot tumours (FDR < 0.1, Fig. 5d). Of these, prominent examples included amplifications targeting the epidermal growth factor receptor (*EGFR*) (7p11.2) and *MYC* (8q24.3) and deletions at 10q23.31, encompassing the *PTEN* tumour suppressor gene in cold tumours and *JAK2* (9p24.1) amplifications in hot tumours. Some of these candidates already have known associations with immune evasion; *MYC* has been linked to an immune evasion phenotype that is amenable to targeting through gene–body demethylation^51^ and *PTEN* deletion has recently been linked to the failure of immunotherapy and decreased cytotoxic T-lymphocyte infiltration in patients and in a mouse model of melanoma^43,52,53^. Among the genomic alterations we identify (for a full list of predicted driver events see Supplementary Data 8), it is likely that some establish, while others are selected for, in different immune microenvironments. In either case, alongside *PTEN* deletion, these alterations warrant further investigation as candidate genomic markers for response to ICB. The enrichment of *EGFR* and *MYC* amplification, together with *PTEN* deletion in cold tumours pan-cancer was striking given the co-expression module linked to increased tumour cell glycolysis and immune evasion in HNSCC, which includes *EGFR* and in which pathway analysis also predicts increased c-MYC and mTORC1 activity^17^. A similar relationship has been observed in triple negative breast cancer^54^ and we therefore investigated this relationship further; initially interrogating the link between *EGFR* protein levels and TILs in two HNSCC cohorts (*n* = 518)^19,28^ we found that samples classified as EGFR high and moderate were significantly more likely to be TIL low than EGFR low cancers after accounting for anatomic site and HPV status (Fig. 5e, *p* < 0.05 and 0.01 for EGFR moderate and high cancers, logistic regression). The positive correlation between EGFR levels (which are themselves correlated with EGFR phosphorylation (activation), Supplementary Figure 3) and the glycolytic signature is maintained across TCGA when matched RPPA profiles and RNA-seq data are compared (Fig. 5f). Notably, the glycolytic signature is enriched in progressing melanomas after PD-1 blockade (Fig. 5g, *p* = 0.06, *t*-test, *p* = 0.02 when excluding stable disease) and is inversely associated with expression of the hot transcriptional signature (Rho = −0.44) that is associated with ICB response (Figs. 4 and 5h).

## Discussion

Of the methods developed to deconvolve cell mixtures into multiple cell types from methylation data, there have not been, to the best of our knowledge, exhaustive studies employed across cancer types. Methods such as LUMP and the Leukocyte Methylation Score estimate only the overall leukocyte fraction, while methods based on expression data either produce relative estimates of abundance within the immune fraction or enrichment scores (CIBERSORT, TIMER) or perform low resolution deconvolution (ESTIMATE)^13,14,21–23^. Combining methylation-based feature selection from both stromal and cancer cells with the robust performance of CIBERSORT previously displayed on gene expression microarray data^14^ allowed us to derive estimates for different infiltrating cell populations as a fraction of the overall sample. We see MethylCIBERSORT and CIBERSORT as complementary tools for studying the tumour microenvironment in cases where both DNA methylation and gene expression data can be obtained, in addition to serving as alternatives in cases where only one data type is available.

While approaches using RNA-sequencing or other transcriptional profiling, such as the construction of an index of cytolytic activity, have been useful in predicting immunotherapy response^7^ and in identifying the role of mutations in genes like *CASP8* in immune evasion^29^, the deeper level of deconvolution made feasible using DNA methylation data allows the roles of distinct cellular subsets and their interdependencies to be dissected. Here, by applying the method to HNSCC, which is marked by a great degree of clinical heterogeneity, we identified lymphocyte-rich and stromal-rich prognostic subgroups consistent with those discovered previously using a variety of independent methods^19,20,24,28,55–57^ and derived insights into the microenvironmental alterations that might be relevant for prognosis. In the process, we showed that our scheme for classifying cancers correlates with well-established immune metrics such as cytolytic activity, neoantigen/mutational load and CTL:Treg ratios. We demonstrated that tumours similar to the HNSCC immune-hot subgroup exist in varying fractions across the vast majority of cancer types. The congruence of our classification with the aforementioned metrics is maintained throughout and translates to broader TCR responses which presumably drive the greater depletion of neoantigens seen in the hot tumours. Combining the accuracy and ability to determine absolute cell fractions of MethylCIBERSORT with the high resolution afforded by CIBERSORT enabled detection of a skew towards antitumour T-helper and macrophage phenotypes in hot tumours, reinforcing our hypothesis that this cluster is enriched for active antitumour immune responses. Consistent with this, we also showed in the limited post-treatment cohorts with gene expression data, that melanomas responding to ICB (particularly anti-CTLA4) display a gene expression signature derived from our hot cluster. Our ICB response analysis also indicates that hot/cold classification based on cellular composition is a superior predictor of response than total mutation or predicted Class I neoantigen loads (both previously proposed ICB response biomarkers), however none of these metrics displayed sufficient accuracy to be of clinical use. Given the complex interplay between tumour genomics, epigenomics and anti-tumour immune responses, it is likely that prediction algorithms incorporating multiple biomarkers such as these, in addition to checkpoint protein expression and spatial information on immune infiltrates (e.g. Immunoscore^58^) will be required for patient stratification.

Genomic analysis identified significant enrichment for events that confer resistance to T-cell-mediated destruction in hot tumours as well as potential sensitisers. Our copy number analysis revealed that *PTEN* deletion, *MYC* amplification and *EGFR* amplification are associated with immune depletion. All three events have been associated with increased glycolysis, which we have previously linked to immune evasion^17^. Our finding that *PTEN* deletion is associated with poor CTL infiltration in this pan-cancer cohort adds substantial support and mechanistic rationale for its proposed role as a determinant of response to ICB. Taken together with the identification of *EGFR* and *MYC* amplification in cold tumours, our analysis suggests that pharmacological inhibition of EGFR/mTORC1/MYC-driven glycolysis could be an effective means by which to ‘warm-up’ these tumours and potentially enhance responses to ICB. The finding that hot tumours frequently harbour functionally-validated immune-resistance mutations offers a potential explanation for the heterogeneity in ICB response even amongst hot tumours (or equally, those with high mutation loads or high cytolytic activity^7^). Secondly, the relative paucity of these mutations in cold tumours (presumably due to the absence of a selection pressure for them) suggests that if we could induce lymphocyte infiltration (e.g. by targeting glycolysis or CAFs^59^), we may improve the effectiveness of checkpoint blockade across a broader range of patients.

Finally, our analysis of neoantigen clonality and immune infiltration patterns adds mechanistic insight to the value of clonal neoantigen burden in predicting response to ICB^5^. In particular, we show that clonal neoantigens are associated with infiltration of CTLs and Tregs, while Th2 cells and CAFs are enriched in tumours with lower clonal neoantigen loads. Why these relationships between neoantigen loads and T-lymphocytes are apparent only when one considers clonal neoantigens is an intriguing question. It could be that since many subclonal neoantigens are expressed by a small minority of cells within the tumour, these evade effective presentation to the immune system. Indeed, in a previous study by several of the authors, it was possible to isolate T-lymphocytes reactive against clonal but not subclonal neoantigens from lung cancer patients^5^. Our data suggest that this is due to a relative paucity of CTLs in tumours with low clonal neoantigen loads and that this is true across a wide range of cancer types.

In summary, the development of a stand-alone method to estimate both tumour purity and stromal composition from DNA methylation data has provided a number of insights that shed light on potential biomarkers for immunotherapy response and the way in which tumour genomes influence, and are shaped by, the immune microenvironment. Beyond analyses of publicly available data such as those we present here, the applicability of the method to both fresh and archival samples should readily allow researchers to explore questions related to the tumour microenvironment and potential therapeutic response across a diverse range of experimental settings.

## Methods

### Development of methylation signatures for deconvolution

Raw data were obtained in the form of IDAT files from the following sources (the number of samples from which each profile was derived is shown in parentheses): granulocytes (12), CD8+ (cytotoxic T-lymphocytes) (6), CD19+ (B-lymphocytes) (6), CD56+ (NK cells) (6), CD14+ (monocyte lineage) (6), eosinophils (6) were from the *FlowSorted.Blood.450k* Bioconductor package^60^. CD4+ cells were removed from the Blood.450k dataset and CD4+ T-cells from the Zhang dataset^61^ (data kindly provided by Dr. Alicia Oshlack) were further divided into FOXP3+ (Tregs) (4) or FOXP3− (6) groups. Fibroblast profiles (4) were from the Gene Expression Omnibus (GSE74877). Neutrophils are the most abundant subset of granulocytes and these samples were therefore aggregated into a single category for further analysis. To generate a DNA methylation signature for cancer cells, we used 450k methylation profiles we previously obtained from a series of six HNSCC cell lines: UM-SCC47; 93VU147T; UPCI:SCC090; PCI-30; UPCI:SCC036 and UPCI:SCC003 (GSE38270, described in ref. ^62^) and additionally those from Iorio et al. (GSE68379)^37^. The files were parsed into R using the *minfi*^63^ Bioconductor package and were normalised using single sample Noob as implemented in *minfi*. To derive signature features, a custom limma-based wrapper function was used to fit a series of linear models for all pairwise comparisons between candidate cell types. Features from this set of analyses were then restricted to MVPs that showed a median beta-value difference of 0.25 at an FDR of 0.01 for that fit or less, with a maximum of 100 MVPs per pairwise comparison. Finally, for use with CIBERSORT, data were transformed from beta values (bound between 0 and 1) to percentages (0–100). Type-wise means were estimated for each probe and cell type and the matrix exported for upload to CIBERSORT.

### Benchmarking using PBMC mixtures

We applied the feature selection pipeline to the matrix of stromal cells that we assembled and then tested performance against 450k profiles of PBMC mixtures with flow-cytometry gold standards. We also applied *LM22* (expression-based CIBERSORT) to datasets consisting of PBMC samples and Follicular Lymphoma biopsies originally evaluated in CIBERSORT^14^. Wilcoxon’s Rank Sum Tests were used to test for differences in correlations with flow-cytometry for cell types and samples, and absolute errors between flow-cytometry and deconvolution estimate. For the expression-based CIBERSORT estimates, we performed comparisons against both calibrated (i.e. enforcing a sum-to-one constraint as reflected in the flow cytometry) and uncalibrated (straight estimates of cell fractions from CIBERSORT) estimates.

### Running deconvolution experiments on HNSCC using CIBERSORT

Data for 464 methylation profiled TCGA HNSCC samples were downloaded in the form of raw IDAT files for the 450k array from the TCGA data. Data were normalised using functional normalisation^64^ in the minfi^63^ package and BMIQ^65^, with 10,000 reference probes for Expectation Maximisation fitting. HPV status was determined using VirusSeq^66^ based on detection of viral gene transcripts.

Beta values for deconvolution associated features, and the signature matrix derived in the previous step, were uploaded to CIBERSORT at https://cibersort.stanford.edu. The data were not quantile normalised due to the potential for global methylation shifts in cancers, and CIBERSORT was run using 1000 permutations. Output files were downloaded as tab-delimited text files and custom parsers were used to import results into R for downstream analysis. FFPE methylation profiles for 42 HNSCC were obtained from Gene Expression Omnibus (Accession GSE38266) using the GEOquery R package, and beta values were BMIQ normalised and analysed using CIBERSORT as described for the TCGA cohort. Wilcoxon’s Rank Sum Tests were used to test for differences in total TIL abundance and TIL subsets.

### Estimating accuracy in tumour deconvolution

In the absence of flow-cytometry-based estimates for the different cell types in the analysed tumours, the estimated fraction of cancer cells from MethylCIBERSORT was compared to sequencing-data based estimates from ABSOLUTE available for 466 HNSCCs from previously published work^22^ using Spearman’s Rank Correlation. Correlations were between ABSOLUTE and other methods of estimating purity/immune cell fraction in this subset of tumours; LUMP, ESTIMATE^23^ and H&E staining assessment of tumour purity (data available in ref. ^22^). Residuals were computed by subtracting the method estimate from the ABSOLUTE value. Distributions were compared using Wilcoxon’s Rank Sum Test. Spearman’s Rank Correlation was used to estimate correlations between expression of marker transcripts and MethylCIBERSORT estimates for multiple cellular populations. Where applicable, multiple testing correction was performed using the Benjamini–Hochberg approach.

### Clustering and correlation analyses

Estimates of immune cell fractions in HPV− HNSCC (HPV-transcript negative) were examined for correlations with other infiltrating cell types using Spearman’s Rank Correlation with BH correction for multiple testing. Clustering was carried out using the clusterCons package with 100 iterations using a Manhattan distance metric. The most robust number of clusters was then selected.

Differences in the distribution of infiltrating cell types by immune cluster were summarised using mean fold changes and tested using Wilcoxon’s Rank Sum Test with BH-correction for multiple testing.

DEGs were identified using limma-trend and were defined at a threshold of a 2-fold change and BHFDR < 0.01. Pathway analysis was carried out using Ingenuity Pathway Analysis, with findings restricted to experimentally confirmed direct interactions in human cells/tissues. Cytolytic activity (CYT) was calculated as the geometric mean of *GZMA* and *PRF1* expression as defined previously^29^. To estimate the contributions of cell population abundances to this, a linear model was fit against log2(CYT) with the different populations as predictors. Wilcoxon’s Rank Sum Tests were used to test differences in CYT and CD8:Treg ratios between the immune clusters.

### Survival analyses

Multivariate Cox Regression was used to estimate the prognostic utility of clusters derived using infiltration patterns with age and stage as covariates. The survival effect of estimated purity was regressed with the same covariates using a Cox regression with coefficients defined per percent increase in purity.

### Genomic correlates

We obtained a list of driver genes inferred by MutSigCV^33^ in TCGA HNSCC cohort from the Broad Institute’s GDAC. GISTIC Copy number estimates thresholded by genes were also obtained from this source. MAF files were obtained from the TCGA data portal. MutSigCV drivers were filtered at a *q* value threshold of 0.01 and mutations in this set were tested for differences in frequencies of occurrence using a Chi-squared test for differences in proportion. Multiple testing correction was carried out using the Benjamini–Hochberg method. Tables of predicted neoantigens were downloaded from The Cancer Immunome Atlas (http://tcia.at).

### Benchmarking performance across other tumour types

Signature features were derived from 450k profiles using the aforementioned heuristic (delta-Beta and FDR cutoffs) with a maximum of 100 features per cell type for a wide range of tumour types, using cell lines allocated to the corresponding tissue in GSE68379^37^ (Table S10) and the aforementioned infiltrating cell types. These signatures were applied to deconvolve methylation profiles and estimates of purity were derived using TCGA samples for which ABSOLUTE, ESTIMATE and LUMP purity estimates were available^22^.

The cell line data were functionally normalised with the infiltrating cell types described earlier before signature extraction was carried out. 450k data for the aforementioned tumour types were loaded from a pan-cancer freeze derived from SAGE synapse for TCGA pan-cancer (syn2812961) and a custom function was used to extract signature probes and generate methylation percentage matrices for deconvolution with CIBERSORT CIBERSORT was run using the graphical user interface [https://cibersort.stanford.edu]. Correlation and residuals analysis were carried out as described above with MethylCIBERSORT purity estimates versus ABSOLUTE, and between previously published methods and ABSOLUTE. Wilcoxon’s Rank Sum Test with Benjamini–Hochberg correction for multiple testing were used to compare distributions, with these estimates sourced from ref. ^22^.

### Pan-cancer analyses of immune cluster assignment

An elastic net model was fit using cellular abundance estimates for HPV− HNSCC using three iterations of 5-fold cross-validation to identify the optimal values of lambda and alpha with Kappa values being the selection criterion. The classifier was then applied to MethylCIBERSORT estimates from 18 further tumour types for which corresponding cancer cell line methylation profiles were available^37^ to allocate immune cluster. Deconvolution was performed as described above and class allocations were made using the elastic net classifier derived from HNSCC.

For immunoediting analyses, we estimated the number of nonsynonymous mutations encoding at least one immunogenic peptide empirically by summing coefficients across each of six base change contexts as well as the number of non-neoepitope nonsynonymous mutations. Together, these were applied to silent mutation counts in each cancer to derive an expected fraction of neoantigens to nonneoantigens. Comparing the observed fraction to the expected fraction yielded the percentage of neoantigens depleted, and using this in combination with the number of observed neoantigens yielded the count of neoantigen-encoding mutations lost specifically to immunoediting. This was then modelled using a negative binomial framework to estimate the influence of immune cluster on immunoediting.

MAF files for mutations were again downloaded from SAGE synapse for all tumours from the MC3 calling effort (syn7214402). Driver mutations were defined based on pan-cancer MutSig analyses previously published^33^ and logistic regression GLMs were used to estimate coefficients for mutation frequencies for immune cluster with tumour type as a covariate. Significant genes were defined at BHFDR < 0.05. Survival analyses were performed using data downloaded from Synapse (syn7343873) using Cox proportional hazards regression with stage and cancer type as covariates. Substages were aggregated into stages and only Stages I–IV were considered. Neoantigen abundance and clonality data were downloaded from The Cancer Immunome Atlas^45^.

Negative binomial modelling was used to model all count data, cytolytic activity was modelled using linear models and binomial GLMs were used to model proportions. Details of covariates, hypotheses and response variables are presented inline. For copy number analyses, SNP6 data were downloaded from the GDC data portal and processed using GISTIC 2.0^67^ on the GenePattern Public Server (arm-level peel off, noise threshold 0.3, FDR < 0.01, driver-gene confidence > 95%) and modelled similarly to mutation data.

### Further resolution of cell types using expression-based CIBERSORT

RNA-seq data were downloaded from the European Nucleotide Archive for the following datasets: PRJEB11844^68^; PRJNA258216^69^; PRJEB5468^70^. Kallisto^71^ was used to quantify gene expression with a reference transcriptome consisting of Gencode Grch37 assembly of protein coding and lincRNA transcripts. Data were then modelled using limma trend and the top 50 markers by *t*-statistics were selected for each cell subset from one versus all comparisons after thresholding with a 2-fold change and FDR < 0.05. These cell types were used to generate a reference profile and CIBERSORT was run to deconvolute samples. For M1/M2 macrophage analyses we used LM22 from the CIBERSORT server as the reference. In both cases, Wilcoxon’s Rank Sum Test was used to estimate differences in distributions.

### Analysis of immunotherapy response

Nanostring data for a panel of immune genes and exome sequencing data were obtained from Chen et al.^42^ and Roh et al.^43^, respectively for patients treated using sequential anti-CTLA4 and anti-PD1 checkpoint blockade.

Clustering and machine learning were carried out using the subset of genes intersecting with the Hot-vs-Cold pancancer signature. .632 bootstrapping was used for hyperparameter tuning and ROC estimation. Negative binomial regression was used to model neoantigen, mutation and subclone numbers, and logistic regression to estimate predictive performance of count data on response.

The number of subclones present in each tumour from the Roh cohort, derived from the EXPANDS algorithm, were obtained from the associated publication^72^. RNAseq data for aCTLA4 pretreatment biopsies were kindly provided by Eliezer Van Allen and genomic data were from the associated publication^7^. Data for post-treatment Nivolumab treated melanomas were obtained from ref. ^44^.

### Validation of EGFR association with cold tumours

RPPA data were downloaded for TCGA cancers from the TCGA portal. IHC data were derived from refs. ^19,28^ for comparison of EGFR protein levels versus TIL levels, previously defined in refs. ^19,28^. ssGSEA scores were used to summarise the activity of the glycolytic gene signature (described in ref. ^17^) and standard statistical procedures were used to assess interrelationships.

### Code availability

Knit R-markdowns of the code used for analysis have been deposited on Zenodo at https://doi.org/10.5281/zenodo.1304766. The MethylCIBERSORT R-package and the signatures we generated are on Zenodo at https://doi.org/10.5281/zenodo.1298968. A Google Group for users of the package can be found here: https://groups.google.com/d/forum/methyldeconvolution.

### Data availability

The data analysed in this study are available either from the Gene Expression Omnibus (accession numbers: GSE35069; GSE74877; GSE38270; GSE68379), the European Nucleotide Archive (accession numbers: PRJEB11844; PRJNA258216; PRJEB5468), SAGE Synapse (accession numbers: syn7214402; syn7343873; syn2812961), The Cancer Genome Atlas Project or from the authors upon reasonable request.

## Electronic supplementary material

Supplementary Information

Peer Review File

Description of Additional Supplementary Files

Supplementary Data 1

Supplementary Data 2

Supplementary Data 3

Supplementary Data 4

Supplementary Data 5

Supplementary Data 6

Supplementary Data 7

Supplementary Data 8
