# Author Correction: Pan-cancer deconvolution of tumour composition using DNA methylation

**DOI:** 10.1038/s41467-018-07155-4

**Published:** 2018-11-02

**Authors:** Ankur Chakravarthy, Andrew Furness, Kroopa Joshi, Ehsan Ghorani, Kirsty Ford, Matthew J. Ward, Emma V. King, Matt Lechner, Teresa Marafioti, Sergio A. Quezada, Gareth J. Thomas, Andrew Feber, Tim R. Fenton

**Affiliations:** 1 Department of Oncology, UCL Cancer Institute, University College London, London WC1E 6BT, UK; 2 Department of Haematology, UCL Cancer Institute, University College London, London WC1E 6BT, UK; 3 Cancer Sciences Unit, University of Southampton, Tremona Road, Southampton SO16 6YD, UK; 4 Department of Pathology, UCL Cancer Institute, University College London, London WC1E 6BT, UK; 5 Division of Surgery and Interventional Science, University College London, London WC1E 6BT, UK; 6 School of Biosciences, University of Kent, Canterbury CT2 7NJ, UK; 7 Present Address: Princess Margaret Cancer Centre, Toronto, ON M5G 2C4 Canada

Correction to: *Nature Communications*; 10.1038/s41467-018-05570-1; published online 13 August 2018

The original version of this Article contained an error in Fig. 4. In panel a, the colour code for hot and cold clusters was inadvertently inverted. In the correct version of panel a, the hot clusters are blue and the cold clusters are yellow. This error has now been corrected in both the PDF and HTML versions of the Article.

