# Supplementary material for: Pan-cancer deconvolution of tumour composition using DNA methylation
Source: Nat Commun. 2018 Aug 13;9:3220. doi: 10.1038/s41467-018-05570-1 (PMC6089972; doi:10.1038/s41467-018-05570-1)
Supplement: Supplementary file 1 — Supplementary Information [file 41467_2018_5570_MOESM1_ESM.pdf]

Pan-cancer deconvolution of tumour composition using DNA methylation

Chakravarthy et al

Supplementary Information

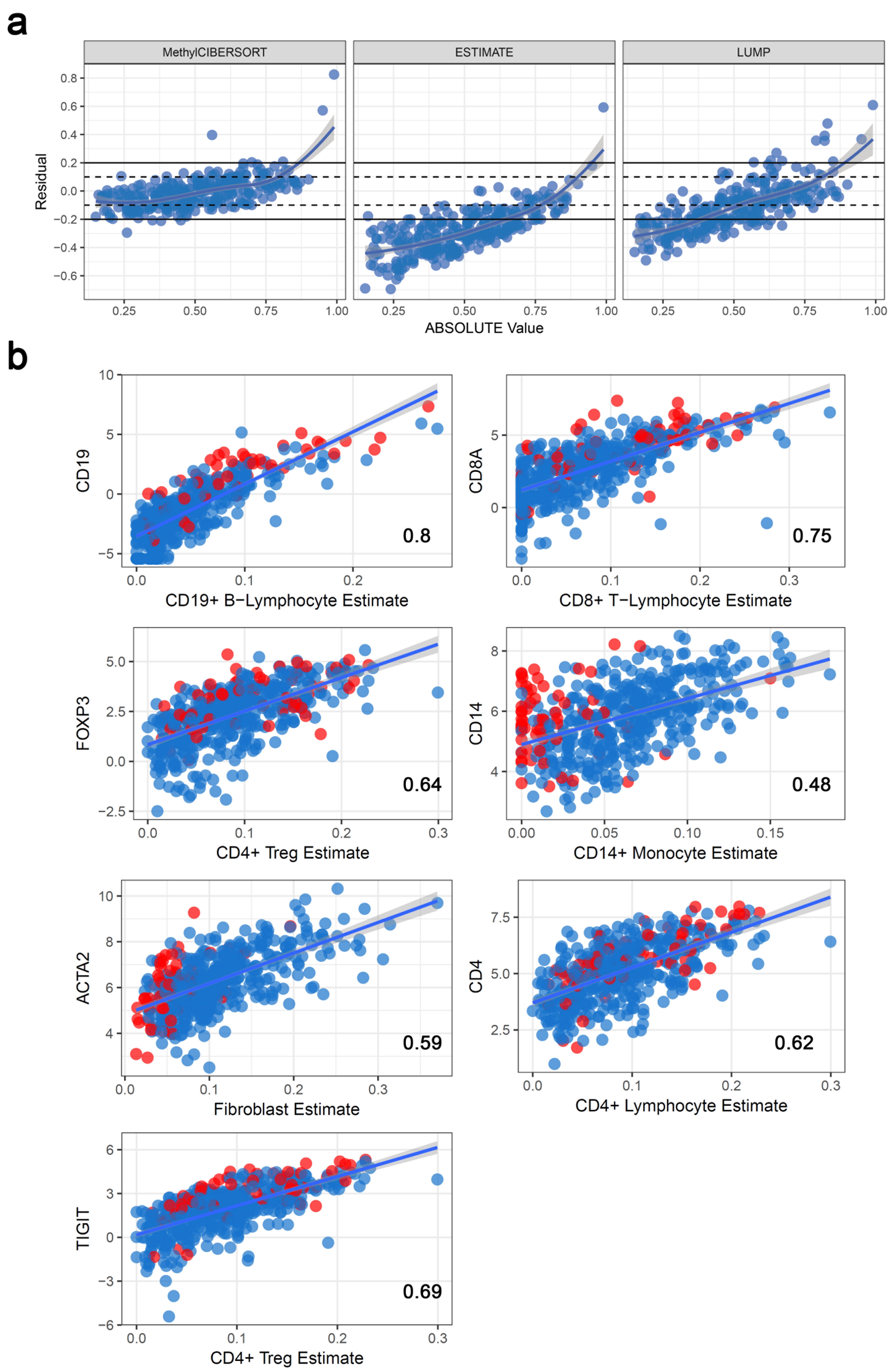

Supplementary Figure 1: (a) Analysis of ABSOLUTE estimate (x-axis) and error from MethylCIBERSORT, ESTIMATE and LUMP in estimating purity in relation (y-axis). **(b)** Correlations (Spearman's Rho) between MethylCIBERSORT estimates and marker gene expression in TCGA HNSCC. Grey areas either side of fitted lines represent 95% confidence intervals.

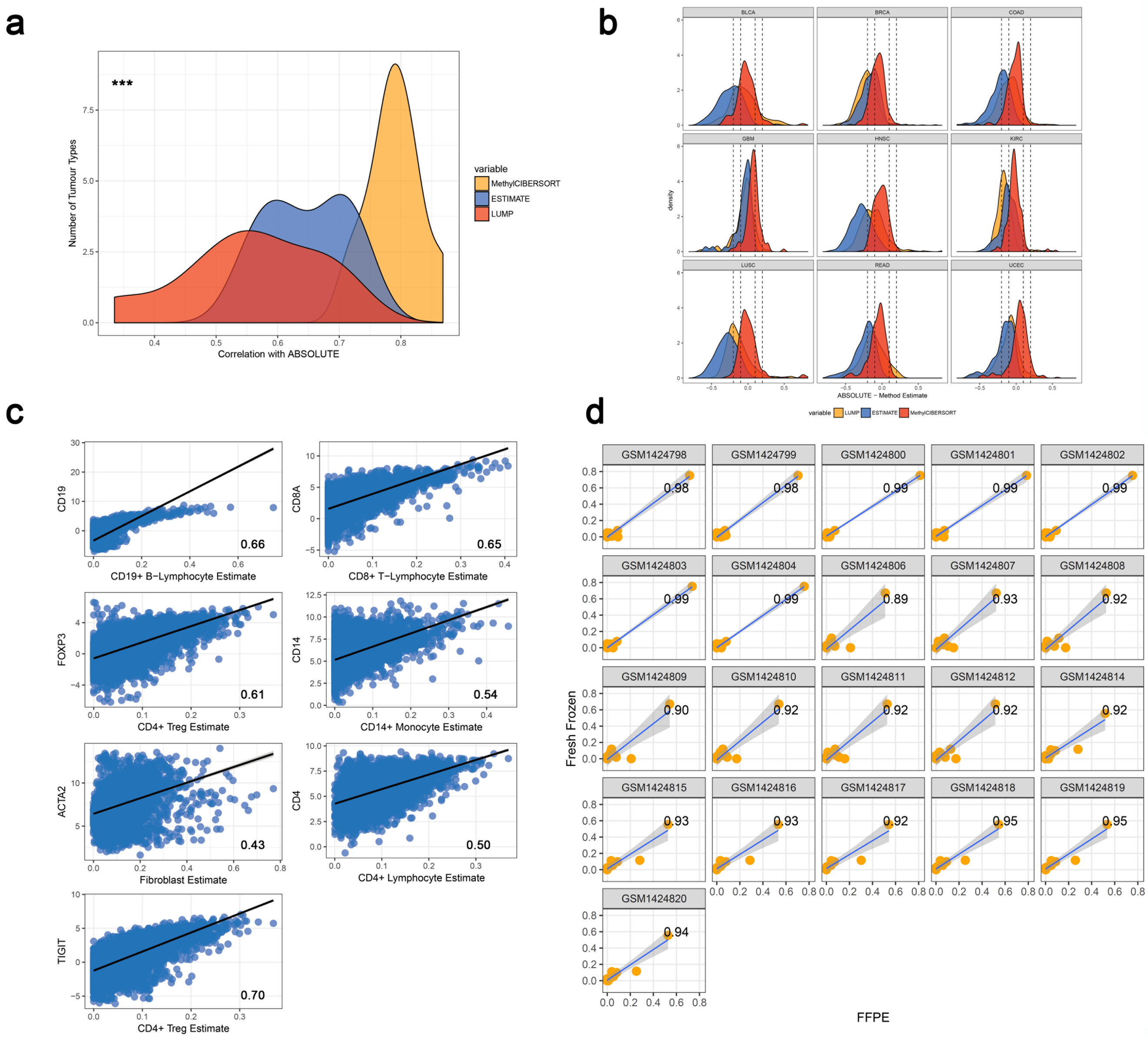

Supplementary Figure 2: (a) Correlation densities for associations with ABSOLUTE purity for MethylCIBERSORT, ESTIMATE and LUMP across tumour types. **(b)** Density plots showing error relative to ABSOLUTE in individual tumour types for MethylCIBERSORT, ESTIMATE and LUMP. **(c)** Marker correlation plots between MethylCIBERSORT estimates and expression of marker genes. **(d)** Correlation plots for 21 450k methylomes relative from FFPE samples relative to their fresh frozen counterparts. Grey areas either side of fitted lines represent 95% confidence intervals.

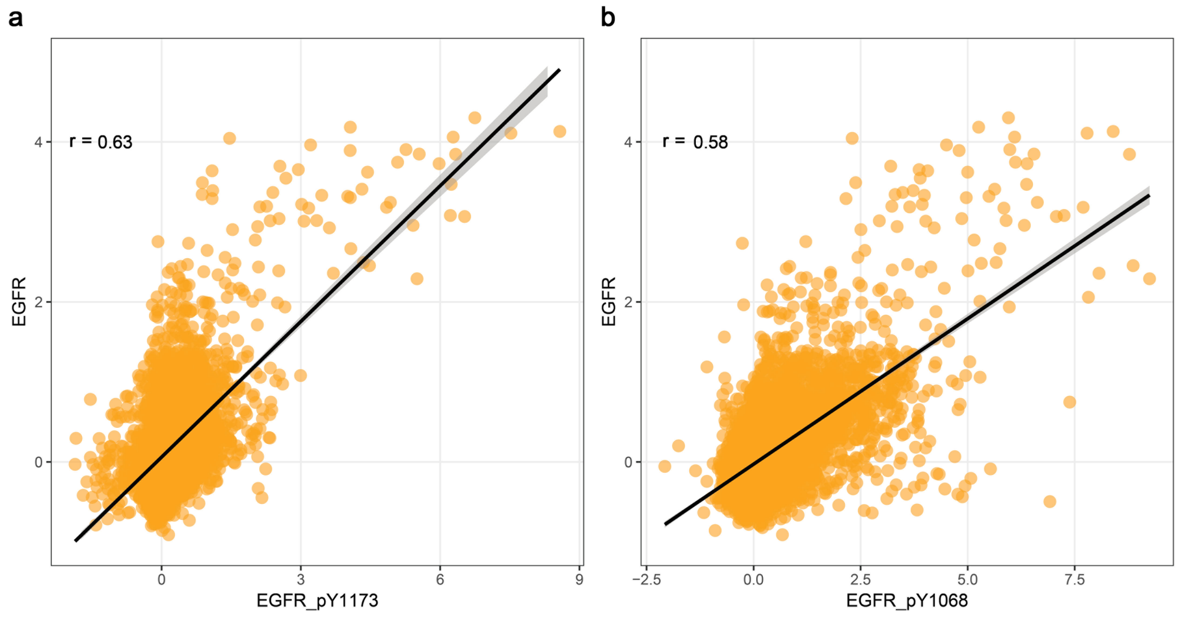

Supplementary Figure 3: Scatterplots showing association between EGFR levels by RPPA and phosphorylation at key activating residues; tyrosine 1173 (a) and tyrosine 1068 (b). Grey areas either side of fitted lines represent 95% confidence intervals. Pearson's correlation coefficients (r) are stated in the panels.
