# Supplementary material for: Pan-cancer deconvolution of tumour composition using DNA methylation
Source: Nat Commun. 2018 Aug 13;9:3220. doi: 10.1038/s41467-018-05570-1 (PMC6089972; doi:10.1038/s41467-018-05570-1)
Supplement: Supplementary file 3 — Description of Additional Supplementary Files [file 41467_2018_5570_MOESM3_ESM.pdf]

Description of Additional Supplementary Files

File Name: Supplementary Data 1
Description: Genes differentially expressed between HNSCC Hot and Cold clusters.

File Name: Supplementary Data 2
Description: IPA canonical pathway analysis for the genes in Supplementary Data 1.

File Name: Supplementary Data 3
Description: Gene ontology analysis for the genes in Supplementary Data 1.

File Name: Supplementary Data 4
Description: Upstream regulator analysis for the genes in Supplementary Data 1.

File Name: Supplementary Data 5
Description: Differential protein expression between hot and cold HNSCCs as measured by reverse-phase protein array.

File Name: Supplementary Data 6
Description: Canonical pathway analysis for pan-cancer hot vs cold transcriptional signature.

File Name: Supplementary Data 7
Description: Results of association analysis between immune cluster and mutation frequency, pan-cancer.

File Name: Supplementary Data 8
Description: Results of association analysis between immune cluster and copy number alteration frequency, pan-cancer.
