# Supplementary material for: Pan-cancer deconvolution of tumour composition using DNA methylation
Source: Nat Commun. 2018 Aug 13;9:3220. doi: 10.1038/s41467-018-05570-1 (PMC6089972; doi:10.1038/s41467-018-05570-1)
Supplement: Supplementary file 2 — Peer Review File [file 41467_2018_5570_MOESM2_ESM.pdf]

Editorial Note: This manuscript has been previously reviewed at another journal that is not operating a transparent peer review scheme. The manuscript was considered suitable for publication without further review at *Nature Communications.*
